# Surgical Suggestions

**Published:** 1907-02

**Authors:** 


### SURGICAL SUGGESTIONS.

Cold abscess and lipoma often simulate each other very closely, especially around the chest. If in doubt aspirate.-

A history of attacks with symptoms of esophageal stricture and intervening periods of well-being is suggestive of cardiospasm.

Have the tracheotomy instruments handy before operating upon a case of angina Ludovici.

In "Ludwig's angina," the cardinal principle in the treatment is extensive incision. An incision that passes no matter how deep into the substance of the submaxillary gland proper, will prove of little avail unless the tissues within the wound have been broken up until they are practically pulpy

In the presence of a hard, diffuse chronic swelling in the neck having some of the appearances of a malignant growth, the possibility that the tumor is a so-called "woody phlegmon of Reclus" must be considered.

All swellings of the lower jaw accompanied by discharging fistula, especially multiple fistulae, shoud be looked upon with the suspicion of actinomycosis until proven to be otherwise.

It is a wise rule to submit all removed hypertrophied prostates to thorough examination by a pathologist. Carcinomatous degeneration may be found in some spot.-

Amputation of a finger gangrenous as the result of a carbolic acid application should not be performed until the line of demarkation is well established. The necrosis may be superficial and in such an instance the finger may be saved by means of skin graft.—*American Journal of Surgery.*

